# Pediatric adenovirus pneumonia: clinical practice and current treatment

**DOI:** 10.3389/fmed.2023.1207568

**Published:** 2023-07-05

**Authors:** Jie Zhang, Yiting Zhu, Yiyang Zhou, Fei Gao, Xinhui Qiu, Jianshun Li, Hao Yuan, Wenwen Jin, Wei Lin

**Affiliations:** Department of Pediatrics, The Second School of Medicine, The Second Affiliated Hospital and Yuying Children’s Hospital, Wenzhou Medical University, Wenzhou, Zhejiang, China

**Keywords:** pediatric, severe adenovirus pneumonia, early recognition, therapy, prognosis

## Abstract

Adenovirus pneumonia is common in pediatric upper respiratory tract infection, which is comparatively easy to develop into severe cases and has a high mortality rate with many influential sequelae. As for pathogenesis, adenoviruses can directly damage target cells and activate the immune response to varying degrees. Early clinical recognition depends on patients’ symptoms and laboratory tests, including those under 2 years old, dyspnea with systemic toxic symptoms, atelectasis or emphysema in CT image, decreased leukocytes, and significantly increased C-reaction protein (CRP) and procalcitonin (PCT), indicating the possibility of severe cases. Until now, there is no specific drug for adenovirus pneumonia, so in clinical practice, current treatment comprises antiviral drugs, respiratory support and bronchoscopy, immunomodulatory therapy, and blood purification. Additionally, post-infectious bronchiolitis obliterans (PIBO), hemophagocytic syndrome, and death should be carefully noted. Independent risk factors associated with the development of PIBO are invasive mechanical ventilation, intravenous steroid use, duration of fever, and male gender. Meanwhile, hypoxemia, hypercapnia, invasive mechanical ventilation, and low serum albumin levels are related to death. Among these, viral load and serological identification are not only “gold standard” for adenovirus pneumonia, but are also related to the severity and prognosis. Here, we discuss the progress of pathogenesis, early recognition, therapy, and risk factors for poor outcomes regarding severe pediatric adenovirus pneumonia.

## Graphical abstract

**Graphical abstract:**
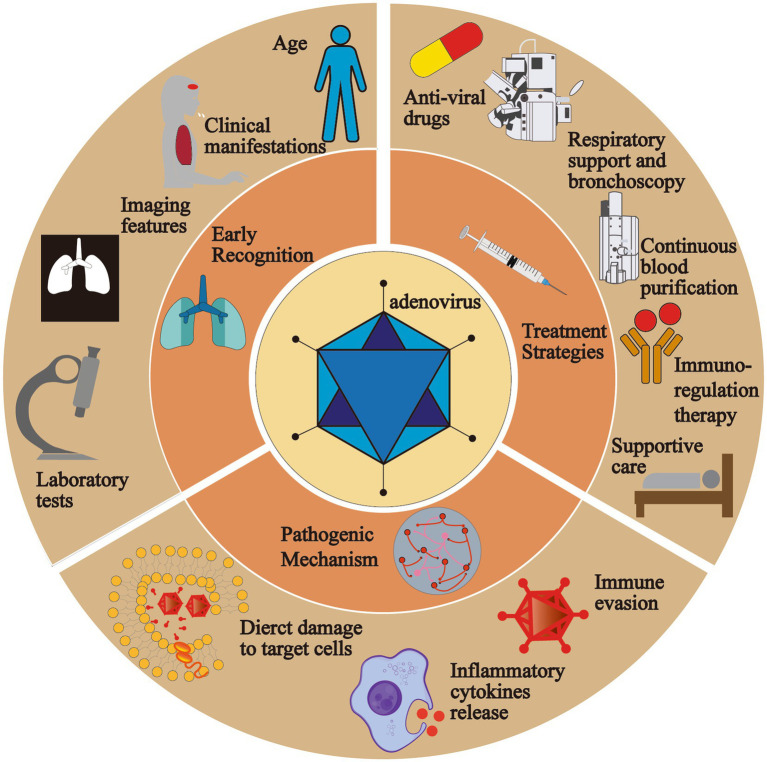
In this review, we lay emphasis on the pathogenic mechanism, early recognition and treatment strategies of severe pediatric adenovirus pneumonia. The pathogenic mechanism of adenovirus is divided into three parts, direct damage to the target cells, inflammatory cytokines release and immune evasion of the virus. Indicators including age, clinical manifestations, imaging features and laboratory tests play an essential role in the early identification. Current treatment strategies are comprised of anti-viral drugs, respiratory support and bronchoscopy, continuous blood purification, immuno-regulation therapy and supportive care.

## Introduction

Adenoviruses are one of the major pathogens that cause CAP (community-acquired pneumonia) in humans, and pediatric adenovirus pneumonia usually happens among those between 6 months and 5 years, accounting for 2%–5% of all respiratory illnesses and 5%–10% of all lower respiratory tract infection in children (1, 2). Unlike other CAP such as Mycoplasma and *Staphylococcus aureus* pneumonia, severe adenovirus pneumonia (SAP) is hard to recognize simply through nonspecific clinical symptoms in the early stage, because the immune system of children is underdeveloped, especially humoral immunity, which clearly explains why the mortality rate is high. It is prone to develop into severe cases along with dreadful sequelae (3). Therefore, we systematically summarize the existing researches and lay emphasis on the pathogenesis, early recognition, treatment, and risk factors for poor outcomes, which are beneficial in reminding clinicians of the roles of the indicators and reducing mortality rate and the occurrence of sequelae. What is more, we also put forward possible directions for future basic and clinical researches on severe pediatric adenovirus pneumonia, especially for therapy, and we wish for more relevant researches carried out to verify their effectiveness, thus enriching treatment options and improving the prognosis of children with severe adenovirus pneumonia (see Graphical abstract).

## Methods

We performed a literature review search on the following databases of academic references and journals: PubMed, Web of Science, Google Scholar, ScienceDirect, Springer, and websites of adenovirus and foundations presenting studies on pediatric adenovirus pneumonia (including the European Respiratory Society website). Articles and reports were initially reviewed by one author, and potential themes related to effective advocacy were identified, which were then reviewed and discussed with all authors. The following keywords were used: adenovirus AND epidemiology, adenovirus pneumonia AND pathogenesis OR mechanism, adenovirus pneumonia AND children AND early identification, adenovirus pneumonia AND children AND treatment OR therapy, adenovirus pneumonia AND prognosis OR risk factors.

## Epidemiology

Adenoviruses are a group of non-enveloped viruses with double-stranded DNA. Since 1953 when it was first discovered by Rowe et al., more than 120 genotypes have been identified that can infect vertebrates, among which human adenoviruses are classified into seven subgenera from A to G (4–6). Moreover, different serotypes of adenoviruses are responsible for different types of disease because the tissue affinity of the virus varies (Table 1). For example, human adenovirus type 1 to 5, 7, 14, and 21 (HAdV-1, 5, 7, 14, 21) are associated with small airway dysfunction, bronchiectasis in children (30), and chronic obstructive pulmonary disease (COPD) in adults (31). Meanwhile, HAdV-8, 19, 37 have been proven to be associated with epidemic keratoconjunctivitis (32). Moreover, SAP and acute respiratory distress syndrome (ARDS) are generally thought to be caused by HAdV-3, 7, 14, 21, 55, which is more common in immunocompromised groups like neonates (33). However, in infected immunocompetent individuals, there are few identifiable symptoms.

**Table 1:** Different serotypes of adenovirus cause clinical manifestations.

Clinical manifestations	Serotypes of human adenovirus	Primary entry receptors	References
Symptoms in the respiratory system	AdV-3,7,14,55,4	CD46, CAR, DSG2	(7–11)
Keratoconjunctivitis	Mostly AdV-8,19,37 and AdV-3,4,7,11,14,53,54 also involved	CAR, CD46, DSG2, sialic acid	(8, 10–15)
Gastrointestinal manifestations*	AdV-40,41	CAR	(8, 11, 16)
Symptoms in the urinary tract	AdV-3,7,21,11,34,35	CAR	(8, 11, 17, 18)
Rare manifestations^Δ^			
OBESITY	ADV-36	TLR	(8, 19, 20)
ENCEPHALITIS	AdV-7,3,2,5	CD46, CAR, DSG2	(8, 11, 21–23)

*Gastrointestinal manifestations include predominant symptoms of gastroenteritis or diarrhea and rare complications of hepatitis (24–26), hemorrhagic colitis, cholecystitis, and pancreatitis (12).

Δ Other rare manifestations include: mononucleosis-like syndromes (27); intestinal intussusception in children (28); viral myocarditis (29); pulmonary dysplasia; sudden infant death.

Infection by adenoviruses can be detected in all four seasons (33). Adenoviruses normally transmit through the respiratory tract, but other routes involve contact and fecal-oral transmission. Surprisingly, it has also been documented that adenovirus can be spread via infected water (34). Serotypes prevalent in different countries differ (35). For instance, in China, HAdV-B3 and HAdV-B7 are predominantly related to SAP (36, 37). However, Yangxi Fu et al. and Hui Dai et al. both demonstrated that HAdV-7-induced pneumonia is more severe than type 3 (7, 36). Meanwhile, the number of cases of HAdV-55 reported is gradually increasing and is expected to become the leading cause of CAP in China (24). However, HAdV-B55 is reportedly responsible for fatal pneumonia in children with a high rate of mixed infection and a greater possibility of inflicting plastic bronchitis after infection (5). In contrast, between 2003 and 2016 in the United States, the US Department of Health and Human Services monitored adenovirus infection, finding out that reported most frequently were HAdV-3 and HAdV-2 (38), while in Latin America, HAdV-7 is the predominant strain of lower respiratory tract infection requiring hospitalization (12).

## Pathogenesis

### Adenoviruses cause direct damage to target cells

**Figure 1:**
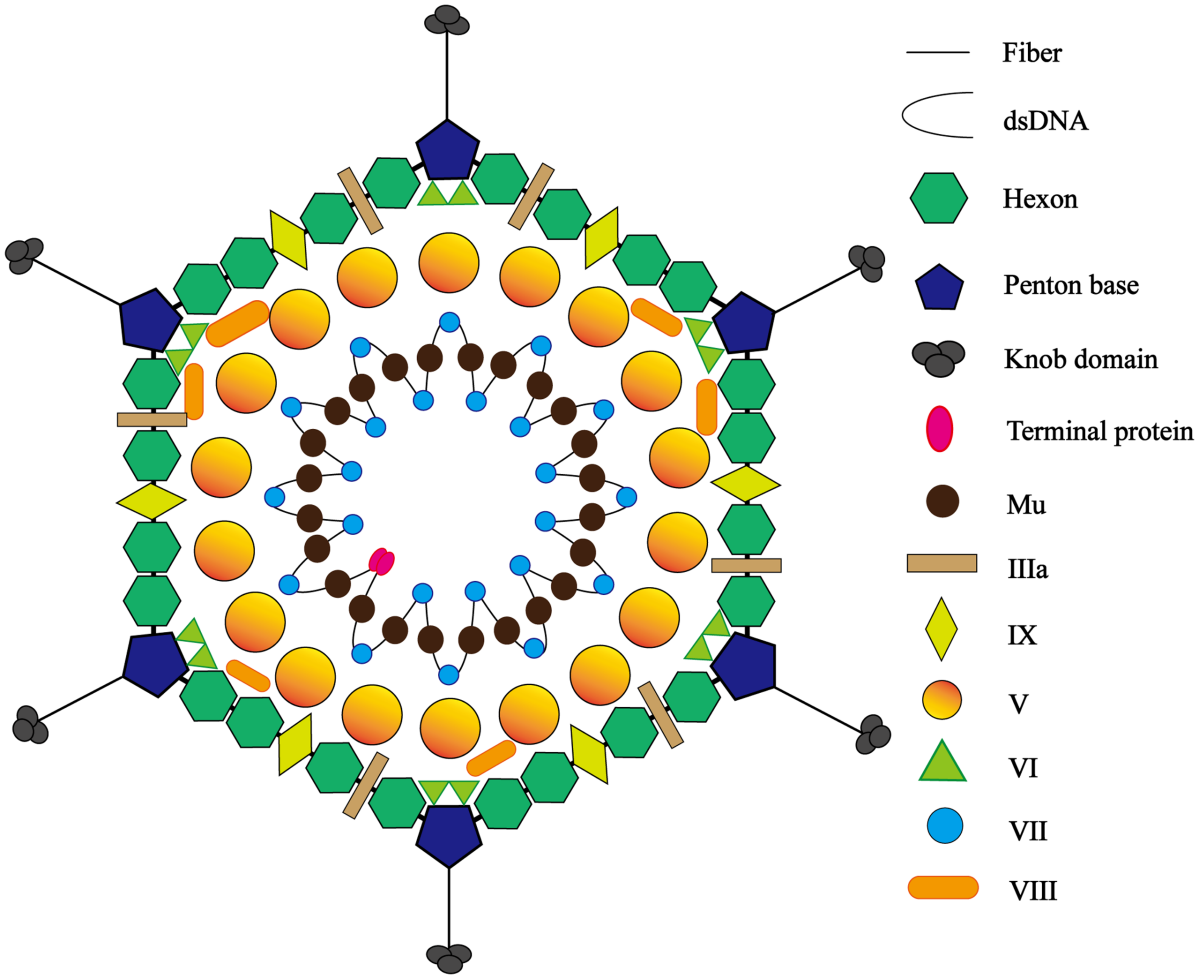
The icosahedral structure of adenovirus. The adenovirus particle comprises two parts, a core and ~90 nm icosahedral capsid. Peripheral icosahedral virus structure consists of three major capsid proteins and four minor capsid proteins, the former involving fiber and knob, penton base, and hexon and the latter including protein IIIa, VIII, VI, and IX. The core contains the viral genome and core proteins and the genome is accompanied by core proteins V, VII, Mu, Tp (terminal protein).

The adenovirus particle comprises two parts, a core and ~90 nm icosahedral capsid. On the one hand, the core contains the viral genome and core protein. The viral genome is divided into early (E), Intermediate (I), and late (L) three regions. The early genome encodes four transcription units, E1 to E4, which are associated with synthesizing progeny viruses in host cells and regulating host immunity. By comparison, L1 to L5, five transcription units in total, are encoded by the late genome concerning synthesis, assembly, and maturation of progeny viruses (39). The genome is accompanied by core proteins IVa2, V, VII, Mu, Tp (terminal protein), and AVP (antiviral protein) (6, 40) (Figure 1).

Conversely, symmetric peripheral icosahedral virus structure consists of three major capsid proteins and four minor capsid proteins, the former involving fiber, penton base, and hexon. The hexon features a serotype-specific antigenic determinant, while the virus relies on the penton base to bind to host cell receptors, mediating the process of cellular endocytosis. The receptors on the cellular surface not only determine what types of cells the virus is allowed to enter, but also play a significant role in how cells respond to viral infection (41). Meanwhile, the fiber contains species-specific antigenic determinants. The four minor capsid proteins, protein IIIa, VIII, VI, and IX, are designed to stabilize the viral capsid and assemble the virus core during duplication (8, 42).

**Figure 2:**
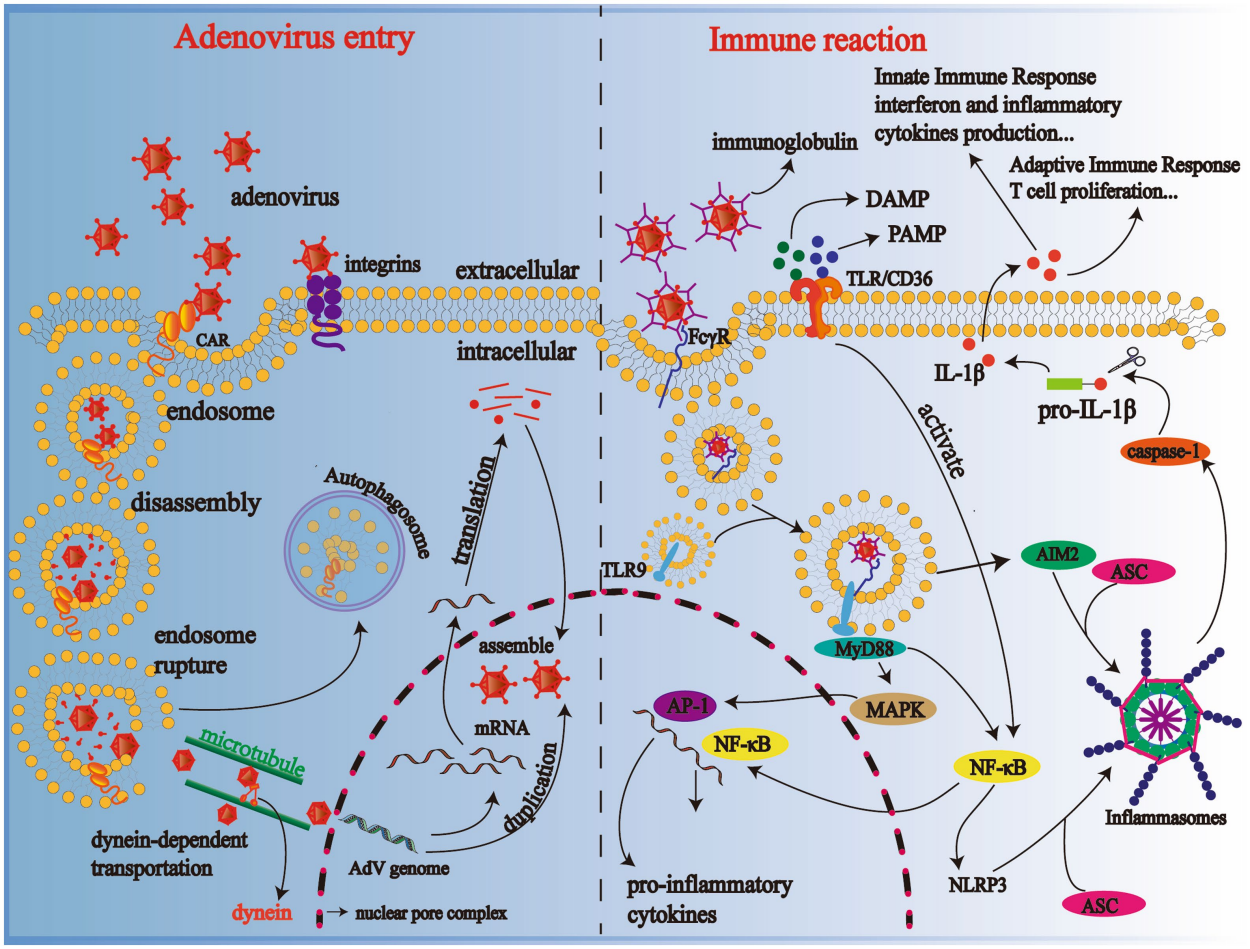
Mechanisms of adenovirus entering the cell and body immune reaction. Progeny adenoviruses are generated through adhesion, cell recognition and endocytosis, disassembly, genome duplication, transcription, translation, and assembly; TLR-binding adenoviruses activate the NF-κB pathway and produce various cytokines in immune cells such as macrophages. CAR, coxsackie-adenovirus receptor; DAMP, danger-associated molecular patterns; PAMP, pathogen-associated molecular patterns; TLR-9, Toll-like receptor-9; NF-κB, nuclear factor-κB; NLRP3 inflammasome, NOD-like receptor family, Pyrin Domain-containing Protein 3 (NLRP3) inflammasome; MyD88, primary response to myeloid differentiation 88; IL-1β, interleukin-1β.

Binding through receptors on the cellular surface, such as coxsackie-adenovirus receptor types A and C (CAR-A, C), CD46/DSG-2 type B and D, and integrins, adenoviruses adhere to the target cells. The location of receptors is a crucial determinant of viral tissue affinity (43). CAR is characterized mainly by respiratory, ocular, and gastrointestinal tropisms, while CD46 is distributed primarily in renal and respiratory cells (43, 44). Subsequently, after adhesion, the phospholipid bilayer of the cell membrane cups inward the virus particles and forms an endosome. The minute adenoviruses bind to the receptors, membrane lytic protein VI is exposed, which converts sphingomyelin in the membrane to ceramide, resulting in endosomal rupture (45, 46). The viruses partially disintegrated in the endosome, are then transported to the nuclear pore complex (NPC) in a kinesin-dependent manner with the help of cellular microtubules, where the viruses perform uncoating and inject DNA into the nucleus (47). Inside the nucleus of the host cell, the viral genome is duplicated and transcribed into mRNA, which is then transferred to the cytoplasm and later translated into viral proteins. Newly synthesized viral DNA and proteins are assembled in the nucleus, producing new viral particles. Finally, the target cell lyses and, at the same time, progeny adenoviruses are released (Figure 2). In the tissue of the alveolar epithelium, if type I alveolar epithelial cells are infected, changes occur in the permeability of the alveolar epithelium and capillaries, meaning a marked increase in exudation and an impaired ability of gas diffusion; similarly, injured type II alveolar epithelial cells lead to a decrease in pulmonary surfactant secretion. Pulmonary surfactant is responsible for pulmonary compliance, and if reduced for various reasons, atelectasis occurs. Therefore, adenoviruses induce pulmonary dysfunction in patients through direct damage.

### Adenoviruses cause indirect damage to the body by activating the immune system

Cytokines, a crucial physiological and pathological agent secreted by immune cells and normal tissue cells make a difference in the regulation of cells and immune injury triggered by pathogens. Adenoviruses are a pro-inflammatory virus that causes the release of inflammatory cytokines in pediatric patients, the levels of which also change in patients with the severity of diseases varying (48). When the human body infects adenoviruses, adenoviruses binding antibodies are recognized by FcγR on the surface of macrophages and dendritic cells and then endocytosed into cells where they bind to Toll-like receptor 9 (TLR9) to activate the MyD-88-dependent pathway and induce cytokine release via NF-κB or MAPK -activating protein-1 (AP) pathway (49–51); scavenger receptor A (SR-A) on the surface of macrophages is also involved in activating the immune response by mediating cellular endocytosis (43). Moreover, pathogen-associated molecular patterns (PAMPs), such as viruses themselves or host derivatives, and danger-associated molecular patterns (DAMPs) derived from hosts combine with TLR/CD36. Then they activate NF-κB, which later excites pyrin domain-containing protein 3 (NLRP3). NLRP3 and absent in melanoma 2 (AIM2) bind to the adapter protein ASC containing caspase recruitment domains (CARD), recruiting and inducing caspase-1 activation, which promotes the transformation from pro-IL-1β to IL-1β by cutting the precursor protein off (52–54). Abnormal release of these inflammatory cytokines after adenovirus infection can lead to apoptosis of alveolar epithelial and endothelial cells, disrupting the pulmonary microvasculature and alveolar epithelial barrier. Finally, there was an increase in vascular exudation, alveolar edema, and hypoxia, even ARDS (Figure 2) (55).

In the late stage of adenovirus infection, alveolar epithelial tissue begins to repair itself when macrophages accumulate in large numbers, secreting piles of growth factors, including epidermal growth factor (EGF) and transforming growth factor (TGF), among which TGF-β is a pleiotropic kind of cytokine, essential for many cellular activities, such as proliferation, differentiation, and adhesion, and is the basic pathogenesis of pulmonary tissue fibrosis (56, 57). Besides, TGF-β could stimulate fibroblast differentiation into myofibroblasts, promote epithelial-mesenchymal transition (51), and induce apoptosis of type II alveolar epithelial cells (58). TGF-β brings about the proliferation of fibroblasts, deposition of extracellular matrix, reduction of pulmonary surfactant, eventually airway fibrosis and narrowing, and even PIBO.

### Adenoviruses evade elimination by the body’s immune system

In the early stage of adenovirus infection, the E1A inhibits NF-κB-dependent transcription, thus suppressing the early inflammatory response, and also inhibits IFN-stimulated genes (ISGs) transcription. Meanwhile, the E1B-19 K protein in the E1B is a potential anti-apoptosis homolog of Bcl-2 (59) and cooperates with the E1B-55 K protein to inhibit E1A-induced apoptosis. Moreover, IFN-stimulated gene expression is inhibited by E1B-55 K, reducing lung inflammation and related cytokine release. When interacting with E4ORF3 expressed by the E4, E1B-55 K can inactivate the MRN complex, which binds to the adenovirus genome and boost viral genome duplication (60). Meanwhile, the E3 interferes with the expression of MHC class I and the NK cell activation receptor NKG2D, weakening the effect of antigen presentation by adenovirus-infected cells and alleviating immune attack mediated by CD8+ T cells (61, 62). Other E3 genes-14.7 K, 10.4 K, 14.5 K, 6.7 K-work jointly to block tumor necrosis factor (TNF)-induced apoptosis and encode R1D1α that inhibits NF-κB activating cytokines and chemokines, downstream of EGFR signaling pathway (63, 64). Proteins of the E4 genes also regulate the splicing of viral mRNA transcribed in host cells, thereby avoiding the formation of dsRNA (65), and thus the activation of protein kinase R (PKR) is prevented (39, 66).

In the late stage, the adenovirus genome enters the nucleus and is transcribed by RNA polymerase III to generate virus-associated RNA (VA RNA), a small noncoding RNA of medium size, which regulates the function of target mRNA such as microRNA (67) and inhibits PKR (13). PKR is a serine/threonine protein kinase that plays an essential role in mRNA translation, apoptosis, and cell proliferation and can inhibit cellular protein synthesis and inflammasome formation. Only when binding with dsRNA, can PKR be activated (68). Additionally, dsRNA competes with VA-RNA to bind PKR, and in the late stage, the efficiency of dsRNA production is high, which means more PKR to inhibit the formation of the NLRP3 inflammasome and downstream cytokine release (41, 69–71).

In conclusion, when the viral load in the body is low, adenoviruses evade elimination by the immune system while entering the cell to proliferate and damage host cells, especially the alveolar epithelial cells; when the viral load is high, the viral DNA is released by the lytic cell and recognized by immune cells such as macrophages, giving rise to the release of cytokines and chemokines in large quantities, even causing an “inflammatory storm.” At this point, immune hyperactivation should take the blame for body function disorder.

## Early recognition

It is known for all that there are no same patients, which means that patients’ manifestations differ. So, the diagnosis of adenovirus pneumonia in children is based on comprehensive information including the patients’ epidemiologic history, clinical symptoms, imaging characteristics, and laboratory tests, among which, virus isolation and serological identification results are “gold standard” but are not suitable for early recognition. Due to the high mortality and terrifying sequelae of SAP, there is no denying that the disease should be recognized early based on clinical manifestations before pathogenic evidence is obtained. In the following, we have summarized the main indicators for early recognition of SAP in children.

### Age

Condition of children’s immune systems of various ages can never be treated the same, so they react differently to viruses. Patients with a poorly developed immune system can be inflicted with persistent viremia. However, a strong immune response is not always advantageous for the body because it can possibly cause an “inflammatory storm,” leading to further multiorgan failure. Studies have shown that children between 6 and 23 months of age are more likely to develop severe pneumonia (7, 72).

### Clinical manifestations

Adenovirus pneumonia has an acute onset, which means that at the beginning, patients can present a fever of 39°C or higher, accompanied by cough and wheezing. The disease course of mild cases is short, and within 2 weeks, fever and respiratory symptoms will vanish normally. If the fever exceeds 2 weeks and the duration is prolonged, the occurrence of SAP should be cautioned (72). Additionally, severe cases manifest severe respiratory symptoms such as respiratory distress, systemic toxicity, and other common systemic symptoms such as abdominal pain, diarrhea, and conjunctival congestion.

In immunocompromised children, who are normally allogeneic stem cell or organ transplantation recipients or patients with inborn immune deficiency, they are better prone to infect with HAdV, owing to deficiency or suppression of immune system. And the manifestations tend to be more severe than in adults, with reported mortality rates occasionally exceeding 50% (67). The initial symptoms usually include fever, enteritis, elevated liver enzymes, and secondary pancytopenia. In solid organ transplantation recipients, the transplanted organ is often the primary target of HAdV, leading to diseases including pneumonia, hepatitis, nephritis, hemorrhagic cystitis, enteritis, and disseminated disease (73, 74). However, in allogeneic stem cell recipients, the spectrum of HAdV-associated diseases is wider, ranging from mild respiratory symptoms to severe manifestations, such as hemorrhagic enteritis, encephalitis, myocarditis, or even multiorgan failure, among which adenovirus-related gastroenteritis is common. It may be the cause of virus dormant in the gut mucosa before transplant (73, 75). As for children with cancer, they usually are immunosuppressed because of chemotherapy and underlying malignancy (74), as a result of which, they are easier to suffer adenovirus infection and prone to develop into sepsis (76). Therefore, additional surveillance of these patients is more than significant.

### Imaging features

More studies have confirmed that the imaging features of adenovirus pneumonia are characterized by pulmonary consolidation rather than interstitial infiltration (77). Specifically speaking, it is common to observe disordered lung markings on both sides, especially the middle and inner lung zone, in the early stage. Along with the development of pneumonia, imaging may present a larger part of lung lobes involved, which is why it is hard to distinguish adenovirus pneumonia and bacterial pneumonia, resulting in misdiagnosis. Furthermore, CT is more sensitive than X-ray in detecting, locating, and measuring lung lesions where the presence of pulmonary atelectasis and pleural effusions is often indicative of severe cases (78, 79).

### Laboratory tests

Currently applied clinical laboratory tests include blood routine (especially leukocyte count), CRP, and PCT. Decreased leukocytes, significantly elevated CRP, and PCT indicate the possibility of SAP, and yet some patients may present a decrease in platelet, evidently an increase in ferritin and lactate dehydrogenase (LDH) (80, 81). Additionally, Na Xu et al. found that LDH, aspartic transaminase (AST), and d-dimer were significantly elevated in critically ill children, suggesting the value of these indicators for early recognition (82); The comparison of TNF-α loci in 320 children infected with adenovirus pneumonia by Shouyuan Zhang et al. using ELISA revealed that the rs3093661 locus A allele, rs1800610 locus A allele, rs3093662 locus G allele, and rs3093664 G locus allele of the TNF-α gene were identified as adenovirus pneumonia susceptibility alleles and were positively correlated with its severity (83); Di-Yuan Yang et al. collected plasma and alveolar lavage fluid of children with adenovirus pneumonia in Guangzhou and compared IgE, dsDNA, and dsDNA-IgE levels, discovering that children with SAP had higher levels of IgE than both non-severe cases and healthy children of the same age. Consequently, they assumed IgE as the result of an overactive host immune system triggered by inflammatory stimuli rather than as a protective factor (84); Yang Shen et al. applied a nomogram to assess the severity of SAP and concluded that CD4 + T cell reduction and elevated IL-6 are reliable predictors of SAP in children (85); It has been illustrated that Tumor Necrosis Factor Superfamily/Tumor Necrosis Factor Receptor Superfamily (TNFSF/TNFRSF) plays a critical role in both local and systemic inflammatory responses in pediatric adenovirus pneumonia and that the TNF/TNFR superfamily receptor-ligand system is highly conserved in almost all mammalian cells and is closely associated with host inflammation, programmed death, and immune cell proliferation and differentiation. TNFSF acts as a co-stimulator to activate lymphocytes and induces pro-inflammatory gene expression through activation of NF-κB (86), of which researchers have observed significantly enhanced expression in patients with adenovirus pneumonia. For one thing, TNFSF13B can influence B cell maturation, proliferation, and antibody class switch and is also involved in the pathophysiology of pulmonary diseases (87). For another, TNFSF14 promotes the synthesis of circulating and intrapulmonary CD8+ T cells, whose expression is enhanced in neutrophils and macrophages in patients affected by HAdV55 (88), further promoting the release of pro-inflammatory agents downstream of the signaling pathway (35). Therefore, TNFSF13B and TNFSF14 may be potential systemic inflammatory markers in pediatric SAP (88).

### Viral load and serological identification

As is mentioned above, SAP is usually associated with HAdV-3, 7, 14, 21, 55, so the severity of pneumonia infected by HAdV can by predicted by serological identification (72), which, in view of availability, is mainly used in scientific researches rather than clinical practice. Instead, RT-PCR can be an alternative to quantify viral load. Leyu Xie et al. and Ruimu Zhang et al. detected and analyzed the adenoviral load in respiratory secretion samples of children with mild and severe adenovirus pneumonia suggesting that SAP samples contained a higher load, and that adenoviral load could be used as a predictor of pneumonia severity (72, 89).

## Therapy

**Figure 3:**
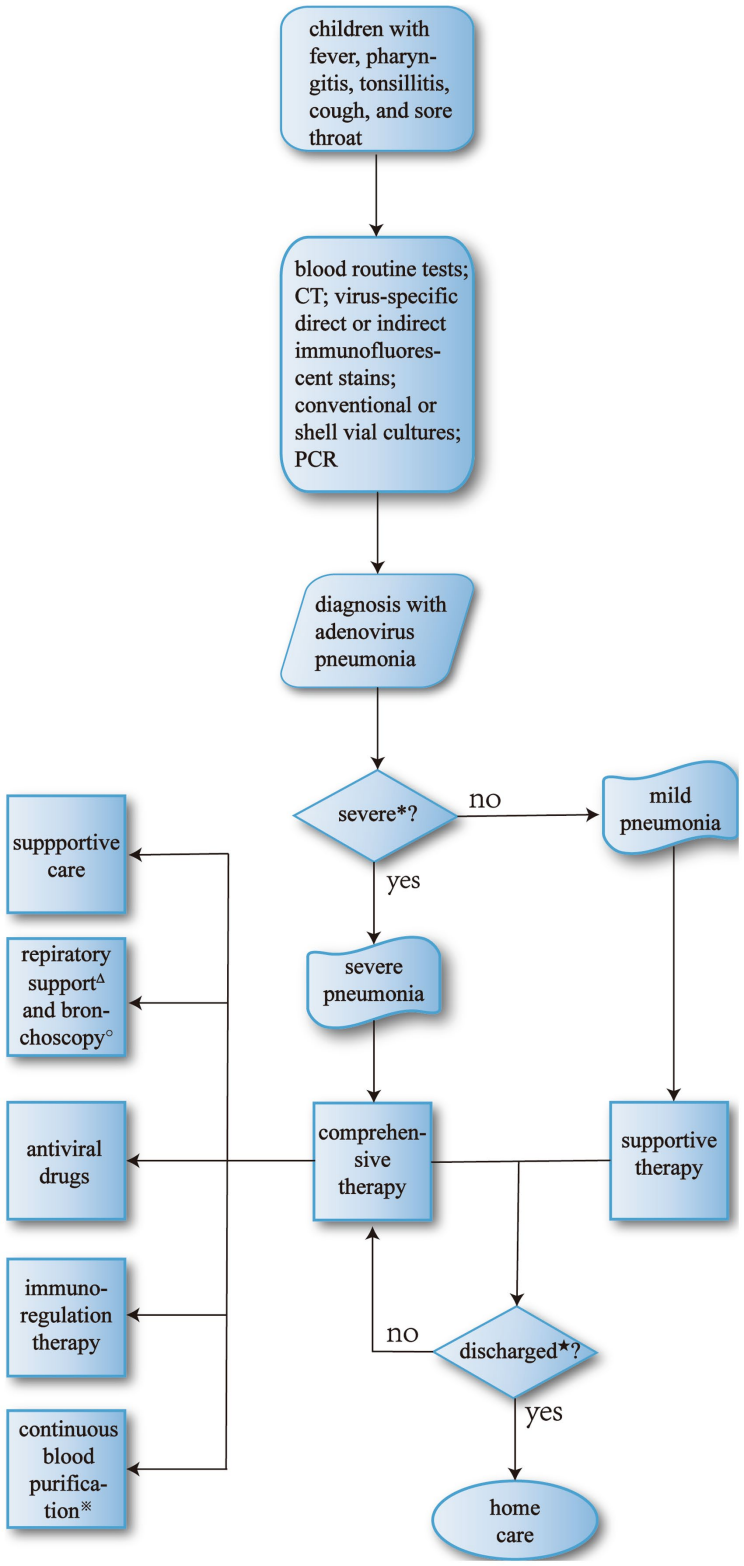
The process of therapy for adenovirus pneumonia. *Major criteria include invasive mechanical ventilation, fluid refractory shock, acute need for NIPPV, hypoxemia requiring FiO2 greater than inspired concentration or flow feasible in general care area; and minor criteria include respiratory rate higher than WHO classification for age, apnea, increased work of breathing (e.g., retractions, dyspnea, nasal flaring, grunting), PaO2/FiO2 ratio < 250, multilobar infiltrates, PEWS score < 6, altered mental status, hypotension, presence of effusion, comorbid conditions (e.g., HgbSS, immunosuppression, immunodeficiency), unexplained metabolic acidosis. Children who have more than one major or two minor criteria should be considered SAP and be carefully monitored (90). ^Δ^The primary indications for mechanical ventilation are: (a) airway protection in a patient who is obtunded or has a dynamic airway, e.g., from trauma or oropharyngeal infection; (b) hypercapnic respiratory failure due to a decrease in minute ventilation; (c) hypoxemic respiratory failure due to oxygenation failure; (d) cardiovascular distress whereby mechanical ventilation can offload the energy requirements of breathing; e. expectant course such as anticipated patient decline or impending transfer (91). ^★^Discharge criteria (90): (a) patients are eligible for discharge when they have documented overall clinical improvement, including activity level, appetite, and decreased fever for at least 12–24 h; (b) patients are eligible for discharge when they demonstrate consistent pulse oximetry measurements of 90% in room air for at least 12–24 h; (c) patients are eligible for discharge only if they demonstrate stable and/or baseline mental status; (d) patients are ineligible for discharge if they have substantially increased work of breathing or sustained tachypnea or tachycardia; (e) patients should have documentation that they can tolerate their home anti-infective regimen, oral or intravenous and home oxygen regimen, if applicable, before hospital discharge; (f) for infants or young children requiring outpatient oral antibiotic therapy, clinicians should demonstrate that parents can administer and children can comply adequately with taking those antibiotics before discharge; (g) for children who have had a chest tube and meet the requirements listed above, hospital discharge is appropriate after the chest tube has been removed for 12–24 h, or if there is no clinical evidence of a chest radiograph, obtained for clinical concerns, shows no significant reaccumulation of parapneumonic effusion or pneumothorax; (h) in infants and children with barriers to care, including concern about careful observation at home, inability to comply with therapy, or lack of availability for follow-up, these issues should be identified and addressed before discharge. ^※^Indications for continuous blood purification include severe sepsis, septic shock; acute respiratory distress syndrome; postoperative persistent hypercytokinemia; severe acute pancreatitis; congestive heart failure, cardiogenic shock; hematological disorders, malignant tumor; thrombotic microangiopathy; trauma, hemorrhagic shock; post-cardiopulmonary arrest; others (92). ^○^The most common indication for bronchoscopy is the presence of signs of airway obstruction. Persistent or severe inspiratory wheezing should be evaluated, if associated with poor weight growth, episodes of apnea, cough while feeding, history suggestive for congenital malformations. Other indications are persistent or recurrent atelectasis, persistent or recurrent localized pneumonia, localized wheezing, or pulmonary hyperinflation, or bronchiectasis, and history suggestive for foreign body inhalation, unexplained hemoptysis. In these cases, bronchial obstruction (e.g., mucus plug, foreign body, or endobronchial tumor), stenosis, compression (e.g., from bronchogenic cysts, vessels, or lymph nodes), anatomical malformation, or bronchomalacia are the most frequent findings (93).

In clinical practice, main treatment basically includes general treatment, antiviral drugs, and respiratory support (Figure 3), which requires physicians to make a comprehensive judgment by examining clinical information such as pediatric clinical symptoms and laboratory tests before formulating a suitable treatment protocol.

### General treatment

A common treatment is fever reduction, isolation, organ function support, and extrapulmonary organ function monitoring, such as the heart, liver, and kidneys. More importantly, children with adenovirus pneumonia should be isolated as early as possible to prevent the occurrence of cross-infection.

### Antiviral drugs

Whether to complement antiviral drugs remains controversial, but the European Respiratory Society (ERS) recommends antiviral drugs in critically ill patients (33). Generally used antiviral drugs are cidofovir, ribavirin, and acyclovir. Cidofovir is a nucleoside and phosphonate analog that inhibits viral DNA polymerase and has broad antiviral ability *in vitro* against various viruses. If applied in the early stage of diseases, clinical outcomes can be positively altered (94). Moreover, its antiviral activity *in vivo* has been documented, but its nephrotoxicity and toxicity to bone marrow have limited its widespread use (12, 35). Another common drug, ribavirin, a guanosine analog, has been reported to be effective in treating adenovirus infection in some cases (95). However, there has not yet been enough evidence to prove its clinical efficacy in adenovirus infection, so it is therefore not recommended (96). Recently, a newly developed drug, brincidofovir, a lipid conjugate of cidofovir, has been reported to have good oral bioavailability compared to cidofovir and powerfully inhibit double-stranded DNA *in vitro*, especially adenoviruses. More particularly, it has low nephrotoxicity and bone marrow toxicity (97). In a recent multicenter retrospective clinical trial, it was used as a preventive therapy for adenovirus infection in children and adults who accepted allogeneic hematopoietic stem cell transplantation, and the results were promising (98). In addition to conventional anti-viral drugs like cidofovir, heparin and its derivatives have also attracted attention as potential antiviral drugs recently. Mark A. Skidmore et al. have shown that chemically modified heparin derivatives could inhibit influenza H5N1 invasion (99) and Kemal Mese et al. confirmed that heparin in the presence of magnesium chloride leads to an enhanced antiviral function on HSV-1 and SARS-CoV-2 (100). Paul S. Kown et al. and Julia A. Tree et al. illustrated that heparin was able to bind to the spike protein of SARS-CoV-2 *in vitro*, inhibiting the activity of the virus (101, 102). More importantly, heparin is able to engage with the two key cytokines, IL-6 and IFN-γand downregulate their inflammatory ability (103), which is the possible mechanism of reducing the inflammatory damage to the body in pediatric adenovirus pneumonia. These studies suggest that heparin may be developed further based on their protein binding and antiviral capabilities and that heparin derivatives may be potential sources of viral inhibitors (104, 105). Nevertheless, since heparin increases the possibility of bleeding, which also restricts its application in adenovirus pneumonia, more clinical trials should be carried out carefully to verify its anti-viral availability and security.

### Respiratory support and bronchoalveolar lavage

Among children with SAP, alveolar epithelial tissue, as well as local ciliary, is altered with increased permeability and exudation and dysfunction of sputum excretion during viral infection, adding that younger children are characterized by a relatively weak ability to cough. As a result, the bronchi and alveoli are blocked because their gas is absorbed, which eventually develops into pulmonary consolidation and atelectasis (79), which, in patients, results in dyspnea. Hence, physicians should aim to restore alveolar function by increasing oxygen supply and removing mucus plugs. Normal respiratory support tools include noninvasive mechanical ventilation, a high-flow nasal cannula (HFNC), high-frequency oscillatory ventilation, and invasive mechanical ventilation. Noninvasive mechanical ventilation is indicated for mild cases of non-dyspnea. In contrast, after noninvasive mechanical ventilation fails, mechanical ventilation is designed for patients with severe hypoxemia, with or without carbon dioxide retention. Since it comes with numerous complications, its clinical application is limited. Nonetheless, early application of noninvasive ventilation in SAP may reduce the use of mechanical ventilation in the late stage (106). A high-flow nasal cannula (HFNC) is a newly emerging oxygen delivery technique recently that provides sufficiently heated and moistened medical gases at a flow rate of up to 60 L/min, which is effective in reducing anatomical dead space, providing constant FIO2 and beneficial humidification compared to other methods of respiratory support (107, 108); on the other hand, HFNC significantly reduces complications compared to invasive ventilation. Historical cohort studies have shown that HFNC reduces the need for intubation, but this has not been confirmed in randomized controlled studies, so further studies are needed to demonstrate its effectiveness (109, 110). High-frequency oscillatory ventilation, known for its small tidal volumes, is widely used in the treatment of ARDS to reduce lung injury during ventilation. However, there is no enough evidence to show that its advantages outweigh the disadvantages (111, 112).

BAL uses pre-warmed 0.9% sterile saline to lavage the selected bronchus by injecting it into the working tunnel of the bronchoscope and then collecting the mixed fluids (93, 113). Bronchoscopy combined with bronchoalveolar lavage can not only clamp the blocked mucus plug out of the small bronchi and effectively ease the clinical symptoms of children with pulmonary consolidation and atelectasis but also obtain alveolar lavage fluid, which is conducive to improving the detection efficiency of pathogens, adjusting drug usage, shortening the course of the disease, and reducing the hospitalization time needed (79). However, some studies have shown that BAL in mild cases can reduce the condition, while there is no significant difference in the incidence of pulmonary sequelae and death cases between the group receiving BAL in the early and late stages of SAP (114).

### Extracorporeal membrane oxygenation

Since it was introduced in 1970, Extracorporeal membrane oxygenation (ECMO) has been used as cardiopulmonary support for patients with respiratory failure. When COVID-19 was prevalent in China, it once played an indispensable role in saving the lives of many critically ill patients (115). ECMO may be considered to help patients with severe pneumonia who do not respond to mechanical ventilation. The advantage of ECMO is low oxygen flow and small tidal volume, which minimizes ventilator-induced lung injury and increases lung recovery (116). In a study analyzing an international multicenter database, the survival rate of patients with adenovirus pneumonia who received ECMO support was 42%, respectively 13.6% in newborns, and 51% in children (117). Yet, doctors should remember that using ECMO accompanies complications, such as bleeding, infection, pneumothorax, and even increased mortality. Short-term or prolonged ECMO use can also increase mortality (118). The timing of ECMO application also impacts patient survival, with studies suggesting an increased mortality rate in children initiated with ECMO more than 6 days after mechanical ventilation (119, 120), a subject that researchers worldwide still need to explore in clinical practice.

### Blood purification

In children with SAP, the immune system is overactivated, and many inflammatory cytokines, such as TNF-α and IL-6, are released (121), causing systemic multiorgan damage. In that case, blood purification can selectively remove inflammatory cytokines and alleviate the damage to the body, thus suppressing excessive immunity and achieving therapeutic purposes (122). Furthermore, it can improve fluid overload, which manifests itself mainly in adenovirus-induced organ dysfunction (123). It has been shown to cause organ dysfunction and increase morbidity and mortality, and avoiding it in the early stages can reduce the duration of mechanical ventilation (124). Meanwhile, clinical studies remain lacking worldwide, especially in children with SAP.

### Immunoregulation therapy

Immunoregulation therapy consists of intravenous immunoglobulin, glucocorticoids and monoclonal antibodies. Immunoglobulin is a series of immune proteins mainly composed of IgG produced by B lymphocytes, which not only binds and inactivates inflammatory factors such as TNF-α and IL-1 to achieve anti-inflammation, but also plays an immunoregulatory function by blocking intercellular interactions mediated by cell surface receptors (125), but the exact effect needs to be further clarified by clinical research (126). In contrast, glucocorticoids are well known to have anti-inflammatory, immunoregulatory, and antishock effects. Studies have shown that early application can improve the success rate of treatment (127), alleviate the tense need for invasive ventilation, and reduce inpatient mortality and even the incidence of ARDS (128), but unfortunately, along with obvious side effects, such as retention of water and sodium and infection. Therefore, whether and how to apply glucocorticoids in clinical practice still requires comprehensive judgment (128, 129). Additionally, in children infecting adenovirus after hematopoietic stem cell transplantation (HSCT), virus-specific T lymphocyte transplantation is an option, and clinical trials have shown good results (130). As is mentioned previously, inflammatory storm is one of the main mechanisms in severe adenovirus pneumonia, so monoclonal antibodies against cytokines such as TNF-α and IL-6 are also possible therapeutic strategies. During the COVID-19 pandemic, some clinical researchers reported that COVID-19 patients treated with TNF inhibitors have better prognosis (131, 132). However, its application remains still controversial, for the fact that the application of TNF-α inhibitors may increase the risk of bacteria and fungi co-infections and that the side effects of using TNF inhibitors in viral infections is unclear (133).

### High-titer neutralizing antibody therapy

Plasmas from recovered patients are collected to extract specific immunoglobulins and other blood products to neutralize viruses for therapy. Meanwhile, plasma therapy has a repair and colloidal support effect on endothelial cells, which is impaired in critically ill patients (134). A retrospective cohort study conducted by Hongyan Peng et al. demonstrated that the administration of high-titer neutralizing antibodies caused faster recovery of body temperature and reduced mortality (135). High-titer neutralizing antibody therapy has shown efficacy in pediatric adenovirus pneumonia, but its source, optimal dose, and most effective application timing have not been further investigated (136, 137).

### Potential treatment

In addition to the treatments discussed above, there are some other newly-emerging ones worth looking forward to, such as gene editing, umbilical cord blood therapy and Chinese tradition medicine. As is mentioned previously, the progeny reproduction of viruses in host cells depends on the function of the viral genome, which, therefore, can be the anti-viral mechanism of potential therapy. The CRISPR/Cas9 system can recognize and cleave specific sequences in viral genome both with high sensitivity and at a fast speed, and has been introduced in knocking out target genes and knocking in genetic materials at specific sites in various animal models (138). Literatures have shown that the technology has been applied *in vivo* to treat some cancers and genetic defects in human (139). On the other hand, as a precise genome modification tool, CRISPR-Cas9 has broad application prospects in the field of molecular medicine, which means that the technology allows specific targeting of viral genomes within host cells, hence inhibiting viral transcription and replication (140). Currently, the CRISPR-Cas9 system is widely used to target multiple types of human DNA viruses, including herpes simplex virus (HSV-1), EBV, cytomegalovirus (CMV) and human papillomavirus (HPV), but it is not yet applied to adenovirus infection (140, 141). However, there are still many challenges facing gene editing therapy, such as the size limitation of foreign gene insertion, the destruction of host genes after integration and subsequent cancer development, difficulty in mass production (141). Consequently, there is a long way in front before it is widely used in clinical antiviral therapy. In the case of umbilical cord blood, rich in hematopoietic stem cells and various types of immune active cells, it can be induced to differentiate into functional mature cells, leading to complete immune initiation and regulation (142, 143). Shuang Liu et al. reported that they used low-dose umbilical cord blood to successfully treat acute respiratory distress syndrome caused by Pneumocystis carinii pneumonia (143). What is more, traditional Chinese medicine is also one of the potential treatments for severe adenovirus pneumonia. In China, Chinese traditional medicine has been widely used in the treatment of pneumonia as symptomatic therapy, but the clinical trials on the efficacy and safety remain still not clear with a lack of comprehensive review and summary (144, 145).

So far, an agreement has been reached that basic treatment for severe pediatric adenovirus pneumonia comprises supportive care, general treatment and respiratory support, but whether to use antiviral drugs remains still controversial. Nevertheless, in recent years, the role of bronchoscopy and BAL is attracting more and more attention because BAL washes away local virus and inflammatory cytokines and bronchoscopy is beneficial in accurate localization of lesions, which possibly reduces hospitalization time (93, 146, 147). However, since it is an invasive method, parents’ worries and children’s coordination need to be taken into careful consideration, which may restrict its application. What is more, ECMO is not viewed as regular therapy but as a rescuing treatment for the fact that although it saves the lives of severe pneumonia patients, the treatment accompanies with a high risk of complications such as bleeding, pneumothorax, and increased mortality (148). As for immuno-regulation therapy like blood purification and neutralizing antibody, its safety and availability are taken as the first concern and for now, it is not yet broadly applied except in clinical trials or experimental therapy (136). Moreover, it is believed that newly-emerging treatments like heparin and Chinese traditional medicine have a promising prospect but more researches should be carried out to verify their effectiveness and safety in clinical trials.

## Risk factors for poor outcomes

Even after comprehensive treatment is implemented by physicians, it remains essential to maintain vigilance for poor clinical outcomes, including PIBO, hemophagocytic syndrome, and death. There are no existing reliable clinical predictive tools to detect the occurrence of poor outcomes. Related clinical manifestations include comorbidity, apnea, wheezing, lethargy, and irritability (149).

PIBO is a common long-term complication of SAP, a pathological change characterized by persistent airway obstruction, especially small airway involvement, and insensitivity to bronchodilators. First reported in 1901, various etiologies, including infection, organ transplantation, toxic gases, and the like (121), can cause bronchiolitis obliterans. PIBO is particularly common in children and has a high incidence in SAP (150). Previous studies have suggested that invasive mechanical ventilation, intravenous steroids, duration of fever, and male gender are independent risk factors associated with the development of PIBO. Instead, longer hospitalization, fever, dyspnea, and hypoxemia are ruled out as potential risk factors for PIBO (150, 151). Invasive mechanical ventilation may cause lung injury through volumetric trauma, barotrauma, and oxygen toxicity; Intravenous steroids could suppress the patients’ immune system, leading to the occurrence of infection and the spread of existing infection. Zhong et al. reported that patients were more likely to develop PIBO if the duration of fever exceeded 10.5 days (152). Independent risk factors associated with death are hypoxemia, hypercarbia, invasive mechanical ventilation, and low serum albumin levels (151, 153). Furthermore, lactate dehydrogenase (LDH) is an important oxidoreductase enzyme that is widely present in living organisms, including the heart, liver, and lungs. When adenoviruses attack the body directly or indirectly, LDH is released into the bloodstream, leading to lung injury (154).

Moreover, LDH, as a laboratory indicator, is convenient, economical, of low hazard, and able to achieve dynamic monitoring. Additionally, a positive correlation has been confirmed between LDH and the severity of pneumonia (155). Xia Wang et al. used receiver operating characteristic (ROC) curve analysis to determine the precision of serum LDH levels as a predictor of death, and the specificity is 93.4% (151). Beyond these, hypoalbuminemia may be a predictor of a poor prognosis (156). And Li Gu et al. demonstrated that fatal outcomes could be predicted by dynamic monitoring of viral shedding, especially in whole blood and that, if it fails to present a significant downward trend around 2 weeks after disease onset, patients are prone to suffer poor outcomes (157).

## Conclusion

SAP in children has a high mortality rate and serious sequelae. In the early stage, adenoviruses can evade immune system elimination and cause direct damage by proliferating in target cells. In contrast, in the late stage, it mainly activates the immune system and even causes an “inflammatory storm,” impairing target tissues and organs. Early recognition of SAP is difficult due to nonspecific clinical symptoms in the early stage. Currently, it is mainly based on the empirical judgment of physicians and laboratory tests because predictive tools with good sensitivity and specificity lack. In clinical practice, symptomatic treatment is the principal method. Newly-emerging treatments such as blood purification and high-titer neutralizing antibody have just presented availability recently, but there is still a long way ahead. Additionally, this article reviews risk factors for the poor outcomes of pediatric adenovirus pneumonia and alerts physicians to PIBO, phagocytic syndrome, and death, with the expectation of reducing mortality and avoiding possible sequelae.

## Author contributions

JZ, YZhu, YZho, FG, XQ, JL, HY, WJ, and WL made a significant contribution to the work reported, whether that is in the conception, study design, execution, acquisition of data, analysis, and interpretation, or in all these areas and took part in drafting and revising or critically reviewing the article. All authors contributed to the article and approved the submitted version.

## Funding

This study was supported by the National Natural Science Foundation of China (82201902), Zhejiang Province Medical and Health Science and Technology Program (2023RC048), and Wenzhou Science and Technology Plan Project (Y20210008).

## Conflict of interest

The authors declare that the research was conducted in the absence of any commercial or financial relationships that could be construed as a potential conflict of interest.

## Publisher’s note

All claims expressed in this article are solely those of the authors and do not necessarily represent those of their affiliated organizations, or those of the publisher, the editors and the reviewers. Any product that may be evaluated in this article, or claim that may be made by its manufacturer, is not guaranteed or endorsed by the publisher.
